# Single-Stage Reconstruction of Nasal Columella Using Bilateral Nasolabial Flaps

**DOI:** 10.7759/cureus.87408

**Published:** 2025-07-07

**Authors:** Gonçalo Caetano, Mariana Santos, Catarina Pinto, Rui Fonseca, Ricardo Matos

**Affiliations:** 1 Otolaryngology - Head and Neck Surgery, Unidade Local de Saúde do Alto Ave, Guimarães, PRT

**Keywords:** bilateral nasolabial flap, columella reconstruction, single-stage surgery, skin neoplasms, squamous cell carcinoma

## Abstract

The nasal columella is often described as a difficult subunit to reconstruct with a satisfactory aesthetic outcome. The nasolabial flap is considered the preferred method for columella reconstruction. We report the case of a patient with a neoplasm of the columella, proposed for surgical excision and columella reconstruction using bilateral nasolabial flaps in a single surgical stage. After excision of the tumor, the remaining skin of the columella was removed to reconstruct the entire subunit. Following bilateral elevation of the nasolabial flap, debulking was performed. The nasolabial flaps were rotated medially and sutured in a space created at the upper portion of the upper lip skin. The main advantage of the bilateral nasolabial flap technique lies in its ability to achieve complete columella reconstruction in a single surgical stage, thus minimizing the need for multiple interventions and reducing the overall recovery time for the patient. The functionality and aesthetics of the reconstruction were considered satisfactory. The technique of nasal columella reconstruction using bilateral nasolabial flaps in a single surgical stage proves to be an effective approach for correcting complex nasal columella defects.

## Introduction

The nasal columella is often described as a difficult subunit to reconstruct with a satisfactory aesthetic outcome, since the principle of the subunit was introduced by Burget and Menick in 1985 for nasal reconstruction [1]. The challenge arises due to the scarcity of adjacent tissue available for reconstruction, as well as the distinct contour and subtle borders of the columella.

There is a wide range of indications for columella reconstruction, such as defects resulting from ischemic lesions, trauma, tumor resection, vascular malformations, and congenital agenesis/dysgenesis of nasal anatomy [2].

The skin color, subcutaneous thickness, columella width, and transition zones at the base of the nasal columella, nasal tip, and nasal floor are aesthetic properties of the normal columella anatomy that must be addressed in the reconstruction of columella defects. Therefore, it is not surprising that there are numerous different techniques for reconstructing this subunit from a variety of donor sites [2].

Local facial flaps for columella reconstruction were first introduced by Blair and Byars in 1946 [3]. Since then, a variety of techniques have been reported in the literature, often named according to their donor site. Despite this expansion in the repertoire of local facial flaps, the ideal technique remains undefined [4].

The nasolabial flap, or melolabial flap, is a local pedicled skin flap harvested from the nasolabial fold, useful in the reconstruction of the nasal ala, lateral wall of the nose, columella, and oral cavity [5]. It is a random pattern flap that generally receives arterial supply from the angular artery, superior labial artery, and dorsal nasal artery [6]. Various authors have described different techniques, considering it the preferred method for reconstructing both composite and isolated columella defects [7-10]. Typically, reconstruction involves two stages and is performed with a unilateral flap with either a superior or inferior base.

In 2018, Devendra Gupta introduced an innovative single-stage technique for nasal columella reconstruction using bilateral nasolabial flaps, which he described in a video published on YouTube® [11]. However, this surgical technique has not yet been formally described in a peer-reviewed publication. The authors intend to publish this technique after using it to close a structural defect in the nasal columella following excision of a squamous cell carcinoma. Single-stage reconstruction offers advantages such as reduced operative burden, faster recovery, and improved patient satisfaction by avoiding the need for a second procedure.

## Case presentation

**Figure 1:**
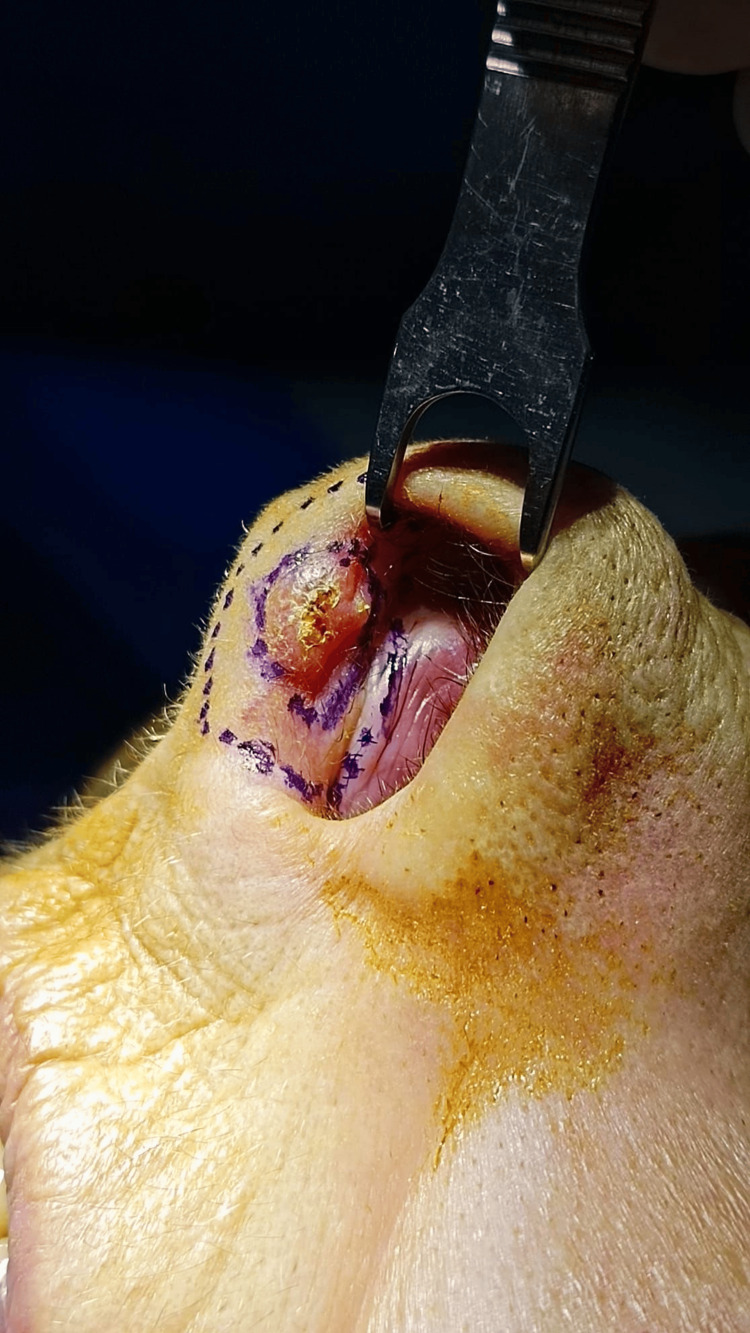
Columella neoplasm suspected to be a basal cell carcinoma

We report the case of an 81-year-old female patient with a history of atrial fibrillation, on anticoagulant therapy, who presented with a nodular lesion on the columella measuring 12×4 mm, with a raised, indurated border, and a central ulceration with crusting. The surface appeared erythematous with areas of scaling, and there were a few telangiectatic vessels visible around the periphery. The lesion was clinically suspected to be a basal cell carcinoma (Figure 1). The lesion was confined exclusively to the columella, with no involvement of the nasal tip, alae, or upper lip. Preoperative assessment confirmed the preservation of all adjacent nasal subunits, allowing for focused excision and reconstruction of the columella alone.

**Figure 2:**
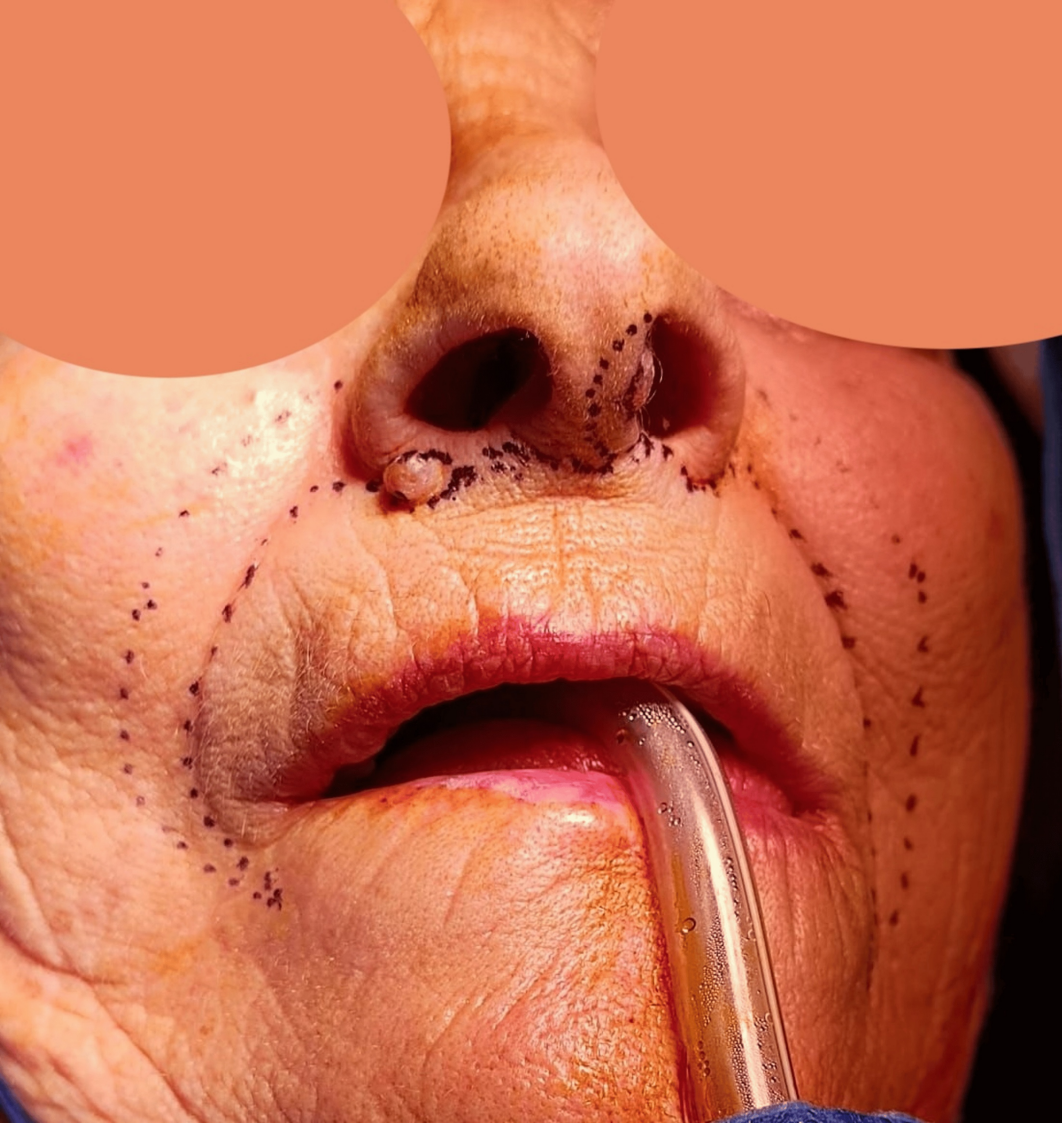
Marking of bilateral nasolabial flap

Surgical excision of the neoplasm was proposed, with columella reconstruction using bilateral nasolabial flaps in a single surgical stage. After marking the nasolabial groove, an inverted Gillies test was performed to ensure sufficient flap length bilaterally. The lower margin of the flap corresponds to the nasolabial groove (Figure 2).

**Figure 3:**
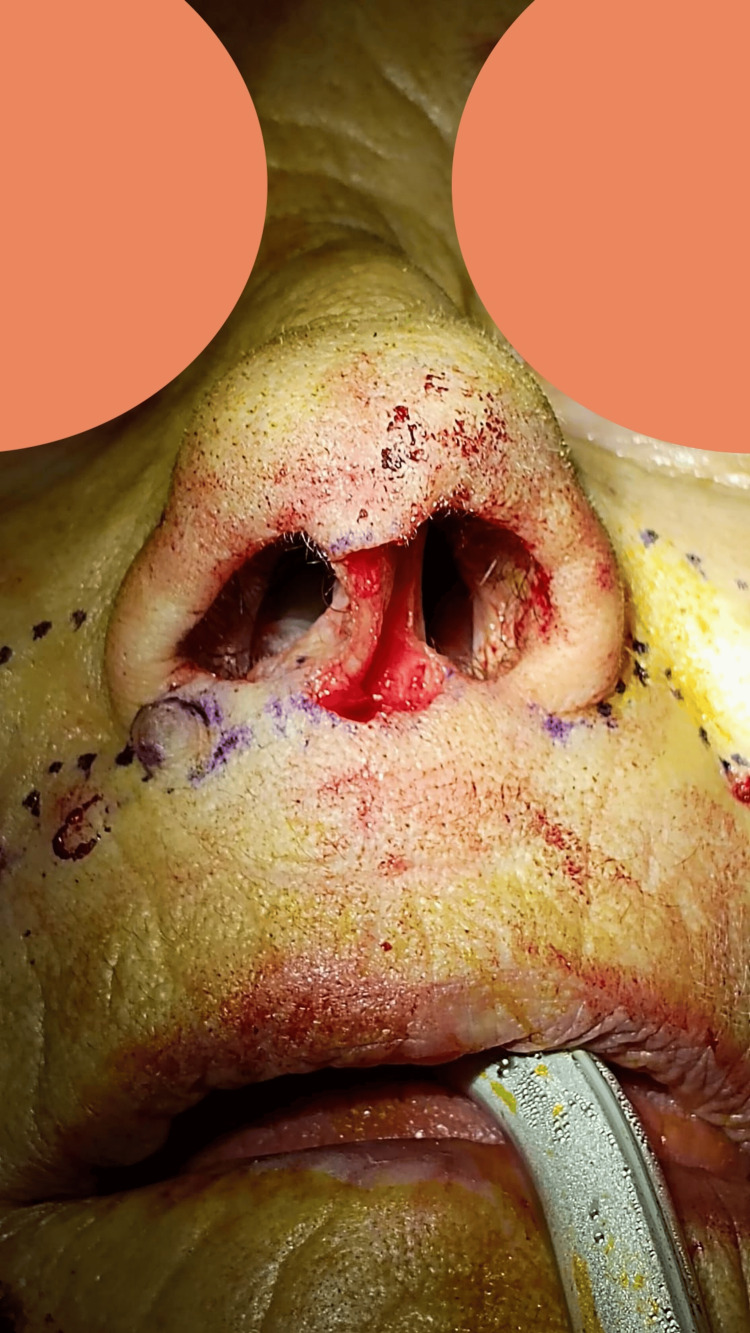
Columellar defect to reconstruct

First, the neoplasm was excised with a 4 mm margin, sacrificing the medial crura of the left alar cartilage and nasal mucosa (Figures 1, 3). The remaining skin of the columella was removed to reconstruct the entire subunit (Figure 3). Subsequently, a cartilaginous strut graft harvested from the septum was sutured to the right medial crura and the left intermediate crura to reconstruct the left medial crura. 

**Figure 4:**
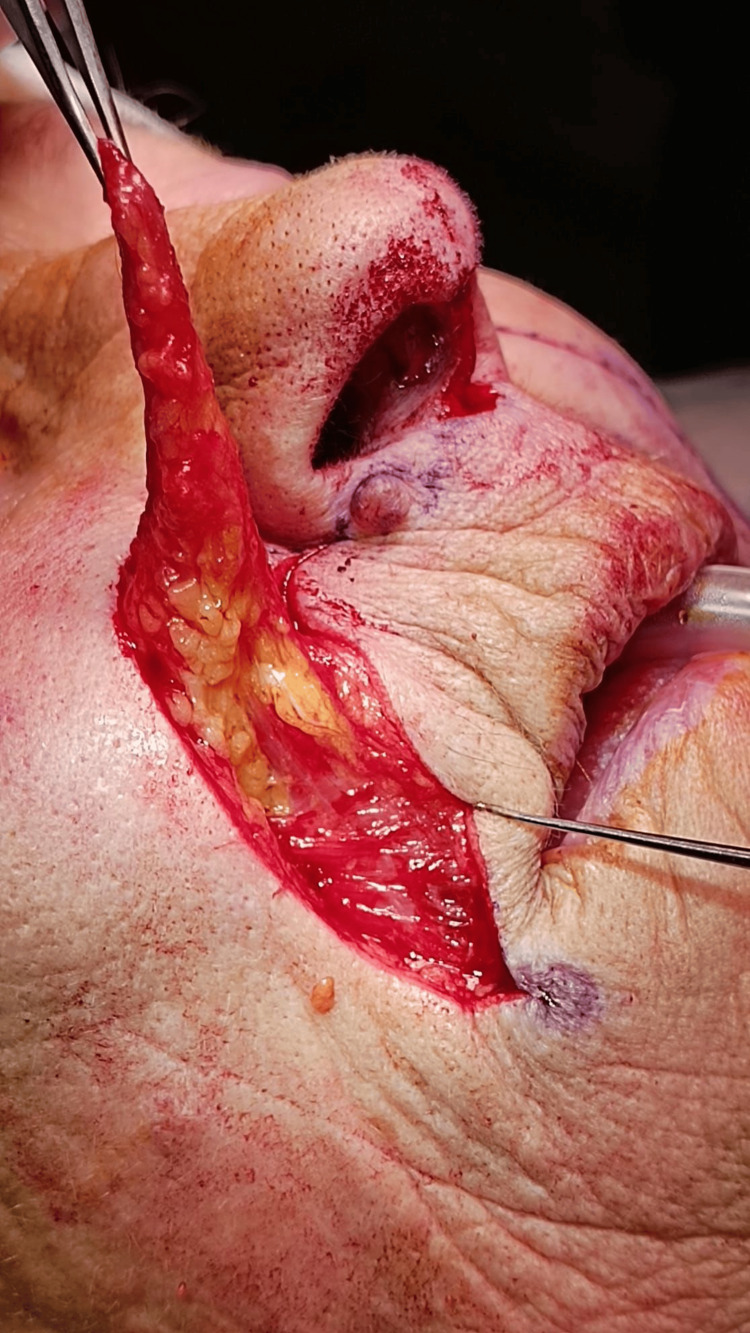
Nasolabial flap elevation: debulking performed to reduce flap thickness

**Figure 5:**
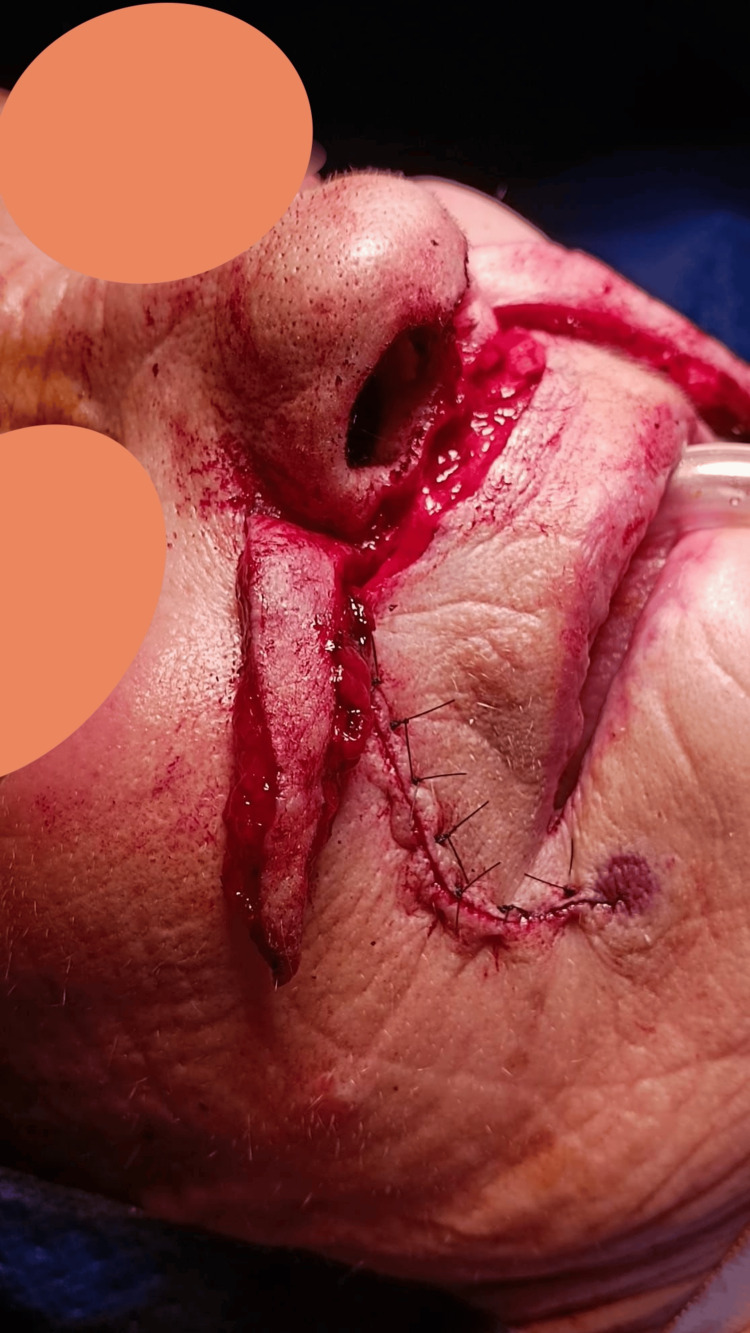
Nasolabial sulcus skin suturing and upper lip skin removal

A bilateral nasolabial flap was raised after subcutaneous infiltration with lidocaine and adrenaline, with elevation performed in an inferior-to-superior direction (Figure 4). Once elevated, the flaps were carefully debulked, preserving a thin layer of subcutaneous tissue. The nasolabial sulcus was then closed using Donati stitches. Subsequently, a skin incision was made between the upper lip and the base of the nasal pyramid, and approximately 2 mm of skin was excised bilaterally from this region (Figure 5).

**Figure 6:**
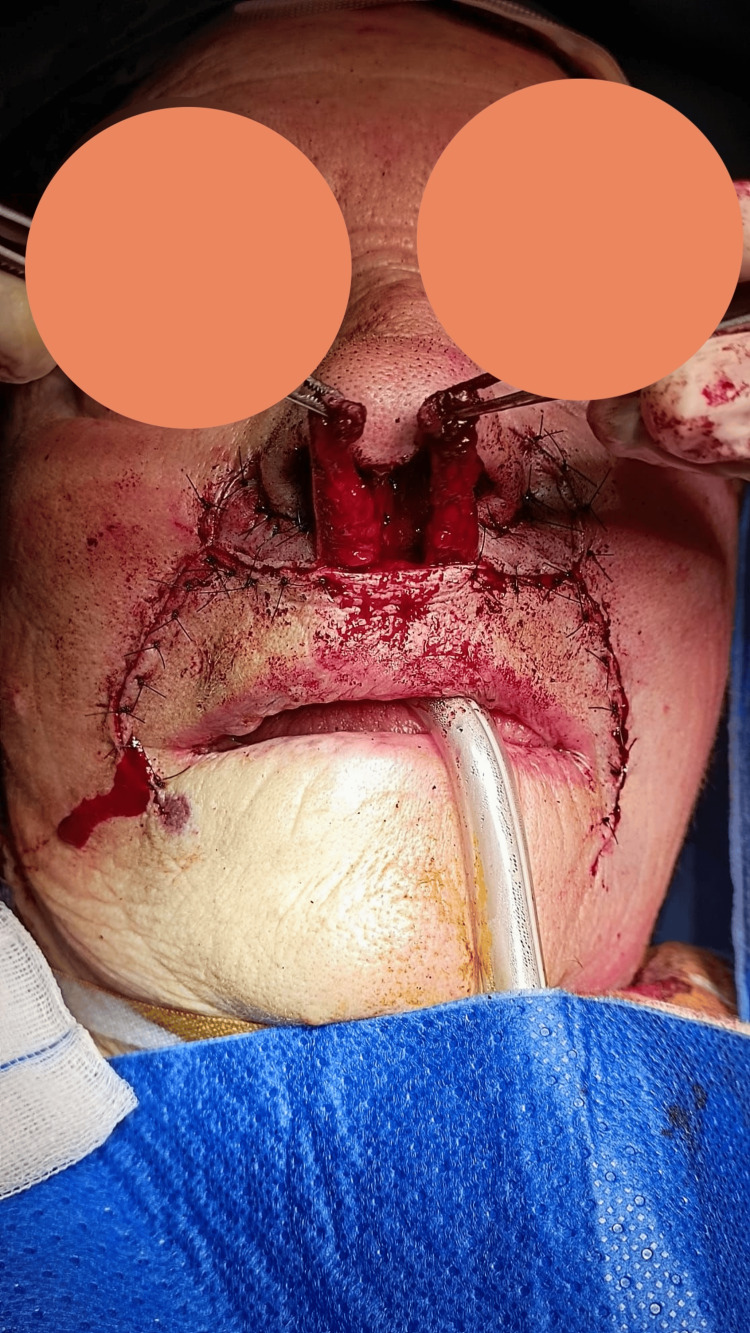
Flap suturing at the upper portion of the upper lip skin

**Figure 7:**
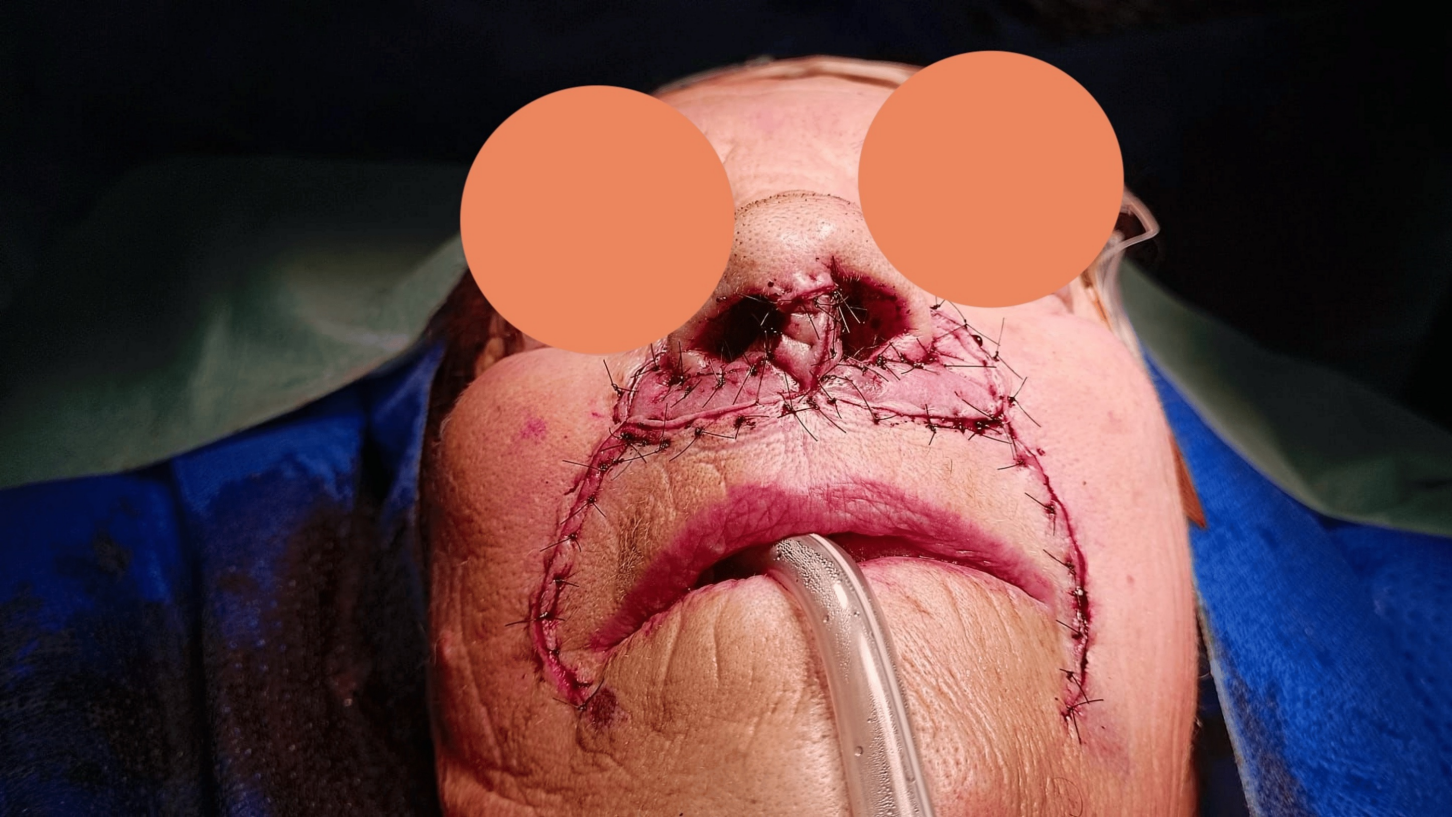
Distal flap suturing

The nasolabial flaps were rotated medially and sutured into the space created at the upper portion of the upper lip skin using Donati stitches once again (Figure 6). Finally, the distal portions of the flaps were sutured together at the midline, sutured to the nasal mucosa laterally, and sutured to the skin of the nasal tip anteriorly with simple stitches (Figure 7).

**Figure 8:**
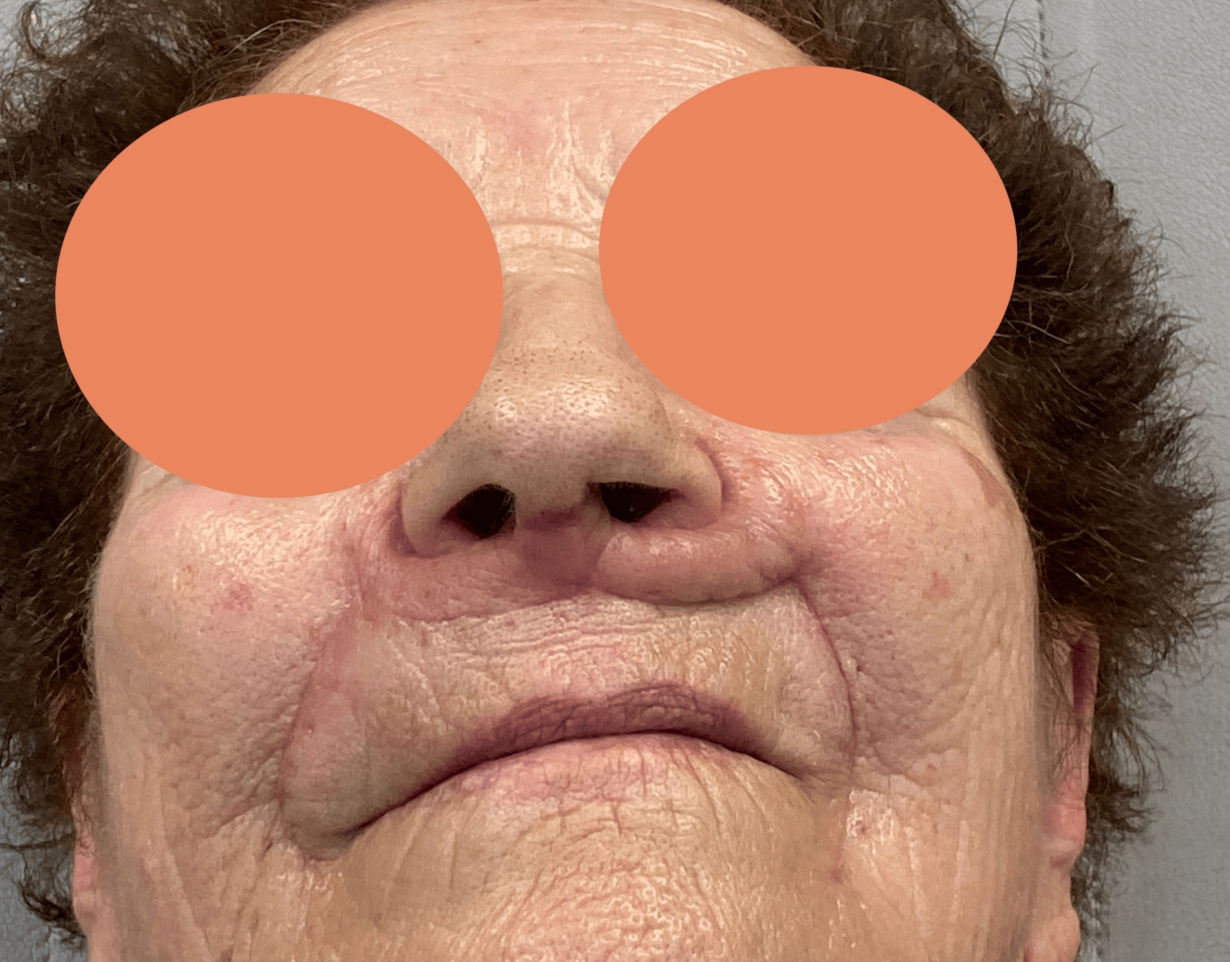
Result after six months

**Figure 9:**
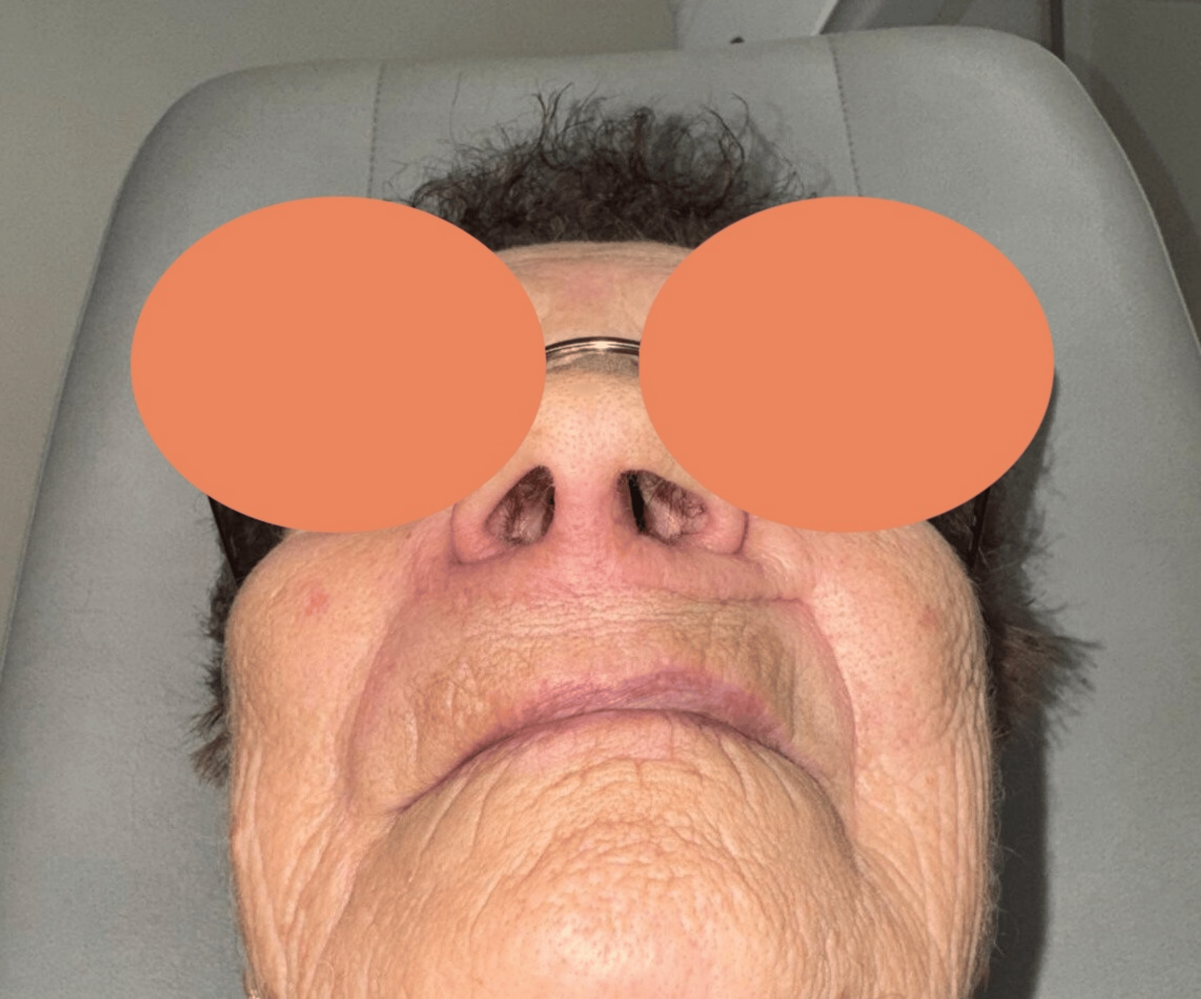
Result six months after secondary debulking (one year after the first surgery)

Given that a septoplasty was performed to harvest cartilage, splints were placed in the nasal cavities, and nasal packing with Spongostan® (Johnson & Johnson, New Brunswick, NJ) was performed. On postoperative day 6, the patient was admitted to the emergency department with epistaxis, which was resolved conservatively, due to a duplication in anticoagulant medication at home. The histological examination revealed squamous cell carcinoma in situ with clear surgical margins. Six months postoperatively, the patient showed good scar healing. However, a pincushion deformity was noted in the left flap, attributed to insufficient initial debulking (Figure 8). A secondary debulking procedure was performed at that time, resulting in partial resolution of the deformity (Figure 9). Functionally, the reconstruction preserved nasal airway patency, maintained nasal tip support and projection, and avoided upper lip stiffness or distortion. No signs of dynamic collapse or external nasal valve dysfunction were noted during follow-up (one year). The patient expressed satisfaction with the aesthetic outcome, and no further surgical interventions were deemed necessary.

## Discussion

Cutaneous squamous cell carcinoma is a malignant tumor originating from epidermal keratinocytes. It mainly occurs in individuals with fair skin and presents as papules, plaques, or nodules that may be smooth, hyperkeratotic, or ulcerated. Surgical excision is considered the first-line treatment for these lesions, with a margin of 4-6 mm [12,13].

Several options for nasal columella reconstruction are found in the literature. The paramedian forehead flap or the unilateral nasolabial flap are the most common [14,15]. The paramedian forehead flap has the advantage of providing ample material for reconstruction; however, the flap may contain hair follicles and requires a second surgical stage [16].

According to Paletta and Van Norman, when a nasal defect consists of total columellar loss, the unilateral nasolabial flap provides an excellent means of reconstruction [17]. In 2014, Ingracio et al. reported that the unilateral superior pedicle nasolabial flap would be the ideal choice for columella reconstruction, considering the preservation of the orbicularis oris muscle [18].

A systematic review and meta-analysis from 2023 showed no statistically significant difference regarding preference of nasolabial or forehead flap for columella reconstruction. The study also reported that there were no statistically significant differences in the risk of complications (partial or total flap necrosis and hematoma/bleeding) between the groups [16].

The single-stage nasal columella reconstruction technique using bilateral nasolabial flaps, as described by Devendra Gupta, offers an innovative and effective approach to addressing complex cutaneous defects in the nasal region. Compared to the surgical techniques typically used in these cases, such as the unilateral nasolabial flap or the paramedian forehead flap, the main advantage of this technique lies in its ability to achieve complete columella reconstruction in a single surgical stage, thus minimizing the need for multiple interventions and reducing the overall recovery time for the patient [2]. Additionally, the use of bilateral nasolabial flaps ensures a large amount of material for reconstruction, as well as good vascularization of the transferred tissues, essential for the viability of the flaps and proper healing. Nevertheless, this patient underwent a second surgical procedure to correct the pincushion deformity.

In 1986, Yanai et al. described a technique for nasal columella reconstruction using bilateral nasolabial flaps in a single surgical stage; however, this technique differs from Gupta's in that the skin is removed from the proximal part of the flap, and it is passed to the nasal region through a subcutaneous tunnel [7].

In the case presented, the patient showed good scar evolution, despite an episode of epistaxis in the immediate postoperative period, which was resolved conservatively. This event was attributed to a duplication in anticoagulant therapy, as the patient was taking both enoxaparin and apixaban simultaneously due to a mistake by a family member, highlighting the importance of strict control of postoperative medication.

The pincushion deformity observed in the upper part of the left upper lip skin is a common complication in reconstruction procedures using local flaps [19]. This deformity occurs due to scar retraction and can be minimized with adequate debulking during surgery. It can also be addressed through debulking in a second surgical stage. However, despite this minor complication, the functionality and aesthetics of the reconstruction were considered satisfactory.

Finally, there is no direct comparison between the new technique and traditional columella reconstruction techniques, such as the unilateral nasolabial flap or the paramedian forehead flap in terms of complications, aesthetic results, and patient satisfaction. Systematic comparisons could provide a better assessment of the relative advantages and disadvantages. This study is limited by a follow-up period of only one year, which may not adequately reflect long-term outcomes.

## Conclusions

The nasal columella reconstruction technique using bilateral nasolabial flaps in a single surgical stage achieved satisfactory aesthetic and functional results in this case, suggesting potential for single-stage columella reconstruction. However, larger studies comparing complications, functional outcomes, and long-term results to established methods are needed to confirm efficacy.

The detailed description of the surgical procedure provides a valuable contribution to the medical literature and can serve as a guide for other facial plastic surgeons facing similar cases. The publication of this innovative technique expands the options available for nasal reconstruction, benefiting patients with similar needs worldwide.
